# l-DOPA and Its Receptor GPR143: Implications for Pathogenesis and Therapy in Parkinson’s Disease

**DOI:** 10.3389/fphar.2019.01119

**Published:** 2019-10-03

**Authors:** Yoshio Goshima, Daiki Masukawa, Yuka Kasahara, Tatsuo Hashimoto, Aderemi Caleb Aladeokin

**Affiliations:** Department of Molecular Pharmacology & Neurobiology, Yokohama City University Graduate School of Medicine, Yokohama, Japan

**Keywords:** l-DOPA, neurotransmitter, G protein–coupled receptor, Parkinson’s disease, dopamine, Lewy bodies

## Abstract

l-3,4-Dihydroxyphenylalanine (l-DOPA) is the most effective therapeutic agent for Parkinson’s disease (PD). l-DOPA is traditionally believed to be an inert amino acid that exerts actions and effectiveness in PD through its conversion to dopamine. In contrast to this generally accepted idea, l-DOPA is proposed to be a neurotransmitter. Recently, GPR143 (OA1), the gene product of *ocular albinism 1* was identified as a receptor candidate for l-DOPA. GPR143 is widely expressed in the central and peripheral nervous system. GPR143 immunoreactivity was colocalized with phosphorylated α-synuclein in Lewy bodies in PD brains. GPR143 may contribute to the therapeutic effectiveness of l-DOPA and might be related to pathogenesis of PD.

## Introduction

Parkinson’s disease (PD) results primarily from the degeneration of dopaminergic neurons in the substantia nigra. l-3,4-Dihydroxyphenylalanine (l-DOPA), a precursor of dopamine (DA), replenishes disease-related lower levels of DA by conversion of l-DOPA to DA in the brain by aromatic l-amino acid decarboxylase (AADC) and alleviates motor symptoms of PD. l-DOPA is mainly synthesized from tyrosine by tyrosine hydroxylase (TH), the rate-limiting enzyme for catecholamine synthesis. Synthetic DA agonists have been included in the treatment of PD for several decades, but l-DOPA is still the most effective therapeutic agent and widely considered the gold standard in PD treatment. However, l-DOPA therapy is associated with adverse effects, such as dyskinesias and impulse control disorders, the underlying mechanisms of which are largely unknown (Ahlskog, 2011; Gottwald and Aminoff, 2011; Rascol et al., 2011; Xie et al., 2015; Ivanova and Loonen, 2016; Voon et al., 2017).

In 1986, we first proposed that l-DOPA itself act a neurotransmitter. l-DOPA is released upon neuronal excitation, and l-DOPA exerts biological activity even with inhibition of AADC to prevent DA formation (Goshima et al., 2014). Most of these responses are antagonized by l-DOPA esters, compounds structurally related to l-DOPA. Recently, GPR143, a G protein–coupled receptor (GPCR), the gene product of *ocular albinism 1* (OA1) (Schiaffino et al., 1999), was reported to have binding activities for l-DOPA (Lopez et al., 2008). GPR143 was distributed in the central and peripheral nervous system and was further confirmed to play a role as an l-DOPA receptor *in vivo* (Hiroshima et al., 2014; Masukawa et al., 2014; Fukuda et al., 2015; Masukawa et al., 2017). GPR143 was also found to be localized in Lewy bodies, the histological hallmark of PD (Goshima et al., 2018). In this review, we revisit l-DOPA therapy and PD pathogenesis in light of l-DOPA action as a neurotransmitter.

## Evidence for l-DOPA as a Neurotransmitter

l-DOPA has been believed to reside exclusively in the cytoplasm of catecholaminergic neurons only as a precursor of the neurotransmitter DA. Contrary to this generally accepted idea, we found that nerve stimulation elicited the release of l-DOPA *in vitro* and *in vivo* experiments (Goshima et al., 1988; Nakamura et al., 1992). The release was dependent on the extracellular Ca^2+^ and sensitive to tetrodotoxin, a voltage-dependent Na^+^-channel blocker. The l-DOPA release was suppressed by inhibitors of P-type voltage-sensitive Ca^2+^ channels and of synaptobrevin (Zhu et al., 2004). These findings together suggest that l-DOPA is released *via* exocytosis (Misu and Goshima, 1993). By using specific antibodies, TH- and l-DOPA–positive but AADC- and DA-negative neurons were shown in some brain areas including the nucleus tractus solitarii (NTS), hypothalamic arcuate nucleus, and magnocellular neurosecretory system (Mons et al., 1988; Misu et al., 1996). Ultrastructurally, l-DOPA–positive signals were localized in the terminals with vesicle-like structures in the lateral habenular nucleus of the house-shrew brain (Karasawa et al., 1992). It is possible that l-DOPA release may arise from l-DOPA–containing vesicles, although vesicular l-DOPA transporter(s), if it exists, have not yet been identified. In addition, exogenous application of l-DOPA produced pharmacological actions in the presence of AADC inhibitor. l-DOPA facilitated the impulse-evoked noradrenaline (NA) release from superfused rat hypothalamic slices (Goshima et al., 1986; Goshima et al., 1991b). Structure–activity relationship study of DOPA-related compounds revealed that D-DOPA, the D-isomer of l-DOPA, did not mimic the effect of l-DOPA. In addition, l-DOPA methyl ester (l-DOPA ME) antagonized the action of l-DOPA in a competitive fashion. Thus, the l-DOPA recognition site(s) has high receptor-like specificity, being stereoselective and requiring specific structural features including the catechol moiety in addition to amino and carboxy groups. These findings support the existence of specific molecular recognition site(s) or receptor(s) for l-DOPA.

Previous reports suggested a role of l-DOPA as a neuromodulator in the regulation of motor function. Certain l-DOPA actions were observed only in PD model but not in normal control animals (Ueda et al., 1995a; Ueda et al., 1995b). Using *in vivo* microdialysis, l-DOPA decreased acetylcholine (ACh) from the striatum of rats lesioned with 6-hydroxydopamine (6-OHDA), but not from that of sham-operated rats. The l-DOPA–induced decrease was not affected by sulpiride, a D_2_/D_3_ antagonist. These findings suggest that l-DOPA by itself regulates the release of ACh. In addition, l-DOPA–sensitive mechanisms were supersensitized in the PD model (Ueda et al., 1995b). A similar supersensitization to l-DOPA was also observed in quinpirole-induced locomotor activity. In normal rats, a highest dose of quinpirole, a D_2_/D_3_ agonist, slightly increased the total accounts of locomotor activities. Concomitant treatment with noneffective dose of l-DOPA (30 mg/kg) potentiated hyperlocomotion induced by quinpirole. In 6-OHDA rats, a noneffective lower dose of l-DOPA (10 mg/kg) potentiated quinpirole-induced locomotor activities (Nakamura et al., 1994).

l-DOPA probably plays a role as a neurotransmitter of the primary baroreceptor afferents in the lower brain stem. A prominent effect of l-DOPA is its depressor and bradycardic actions when microinjected into the NTS of anesthetized rats (Kubo et al., 1992; Yue et al., 1994; Misu et al., 1996; Goshima et al., 2014). Phenylephrine-induced hypertension triggered l-DOPA release in the NTS and reflex bradycardia temporally associated with the rise and recovery of blood pressure. The l-DOPA release and bradycardia were abolished by denervation of bilateral carotid sinus and aortic nerves, which contained the baroreceptor afferents. Electrical stimulation of aortic nerve released l-DOPA and induced depressor and bradycardic responses, and these responses were antagonized by bilateral injection of l-DOPA ME into the NTS (Yue et al., 1994). The depressor response induced by l-DOPA might possibly be related to orthostatic hypotension, which is often encountered in PD patients on l-DOPA therapy (Connolly and Lang, 2014; De Pablo-Fernandez et al., 2017).

## Receptor Candidates Targetted by l-DOPA: Their Localization and Pharmacology in Normal Conditions

These findings together suggest that there is one or more specific receptors for l-DOPA. l-DOPA did not interact with cognate monoamine receptors (Goshima et al., 1991b; Misu et al., 1996). On the other hand, l-DOPA inhibited specific binding of [^3^H]-AMPA in the brain membrane preparations, and l-DOPA induced current response in *Xenopus laevis* oocytes expressing AMPA receptors (Miyamae et al., 1999). However, l-DOPA interacted with AMPA receptors with a low affinity (ED_50_ of 2.2 mM). In addition, DOPA ME and l-DOPA cyclohexyl ester (CHE), l-DOPA antagonists, did not interact with AMPA receptors (Miyamae et al., 1999). These findings suggest that the l-DOPA CHE-sensitive actions of l-DOPA are mediated through recognition sites other than monoamine receptors and AMPA receptors. A clue to the identification of l-DOPA receptors involved genetic analysis of *oa1*, the gene responsible for ocular albinism type 1 (Schiaffino et al., 1999). The gene product of *oa1* referred to as GPR143 is critically involved in organogenesis of the melanosomes. Based on sequence similarities, the protein could not be assigned to any GPCR subfamily. Although binding activities of both DA and l-DOPA to GPR143 were detected, only l-DOPA induced intracellular Ca^2+^ response in cell lines expressing GPR143 (Lopez et al., 2008; Hiroshima et al., 2014). Dopamine also interacted with GPR143 with Ki of 2.4 µM, a comparable value to that for l-DOPA, but produced no functional response on intracellular Ca^2+^ levels in GPR143-expressing CHO cells. Dopamine may therefore act as an antagonist or inverse agonist against GPR143, which is consistent with the structural features and the homology model for GPR143 (Ghosh et al., 2012). These findings indicate that l-DOPA may be an endogenous ligand for GPR143. The role of GPR143 in mediating the l-DOPA response was then examined in the NTS of anesthetized rats. The shRNA knockdown of GPR143 in the NTS abolished or attenuated the depressor response to l-DOPA but not to glutamate microinjected into the NTS. l-DOPA CHE also suppressed the l-DOPA response and displaced the specific binding of [^3^H]-l-DOPA in CHO cells expressing GPR143. These findings suggest that GPR143 is involved in mediating the l-DOPA actions in the NTS (Hiroshima et al., 2014).

GPR143 is highly expressed in the retinal pigment epithelial cells (Schiaffino et al., 1999), and most studies have been conducted to delineate roles of GPR143 in melanogenesis (Schiaffino, 2010; De Filippo et al., 2017). Using specific antibodies against GPR143 and GPR143-KO mice, we investigated tissue distribution of GPR143. GPR143 immunoreactive cells were localized in the hippocampus, cerebral cortex, striatum, substantia nigra, hypothalamic median eminence and suprachiasmatic nucleus, NTS, and caudal ventrolateral medulla and olfactory bulb (Masukawa et al., 2014). A similar distribution pattern was confirmed in wild-type (*Wt*) mouse brains (Fukuda et al., 2015). Of note, GPR143 signals were observed in areas involving both hippocampal and cortical circuits as well as in basal ganglia and hypothalamic and brainstem neural networks. In line with GPR143 expression pattern, several observations reported an effect of l-DOPA in enhancing performances in executive verbal tasks and visuospatial working memory (Kulisevsky et al., 1996; Costa et al., 2003; Poletti and Bonuccelli, 2013), as well as in increasing secretion of growth hormone or cortisol (Biermasz et al., 2005; Muller et al., 2011; Marakaki et al., 2015), both of which being regulated by neurosecretory hypothalamic nuclei.

The downstream signaling pathways triggered by l-DOPA through GPR143 *in vivo* remain unknown (Schiaffino, 2010). Biochemical and morphological analysis suggest that Gai3, Go, and Gq are potential G protein partners for GPR143. Among them, Gai3 was shown to coprecipitate and colocalize with GPR143 (Young et al., 2008). In addition, the retinal pigment epithelial cells of Gai3-deficient mice contained fewer and larger melanosomes, and analysis of the optic pathways revealed a significant reduction of ipsilateral retinofugal projections, both of which were similar to the phenotypes of *Oa1/Gpr143*-deficient mice (Young et al., 2008). Although overall picture is far from understood (Schiaffino, 2010), it is likely that Gai3 plays a role at least in melanosome biogenesis and in the development of the optic tract.

**Figure 1:**
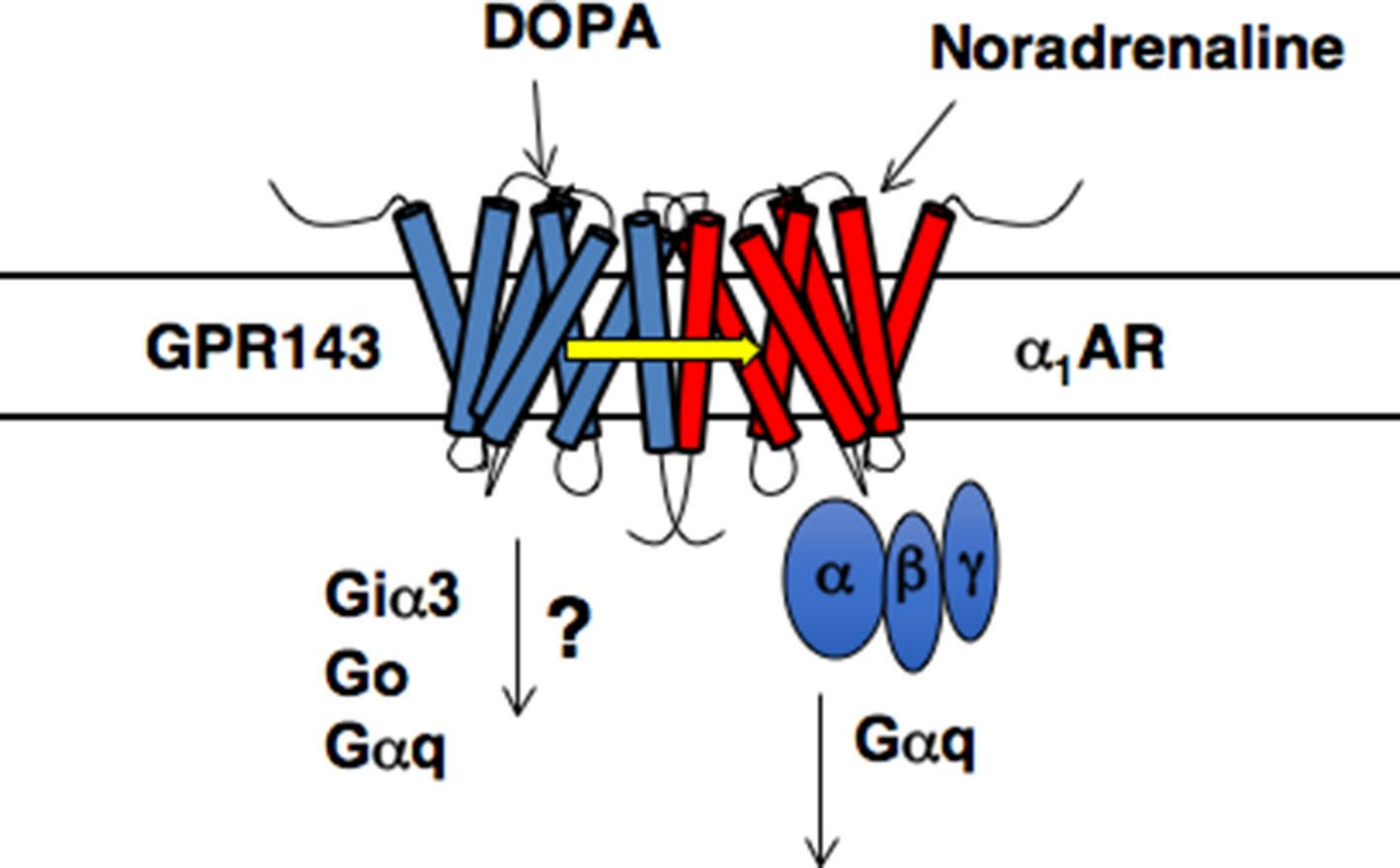
Proposed model of GPR143 and α1_B_AR heteromers (modified from Maurice et al., 2011).

There is additional evidence for the role of GPR143 as an l-DOPA receptor in the peripheral cardiovascular system (Masukawa et al., 2017). Phenotypic analysis of *Gpr143* gene-deficient (GPR143-KO) revealed that the pressor response to phenylephrine, an agonist of a_1_-adrenoceptors (α_1_ARs), was attenuated in GPR143-KO compared to that in *Wt* mice. The pressor response to vasopressin was not altered in GPR143-KO mice. In isolated GPR143-KO peripheral arteries, phenylephrine-induced contraction was reduced, indicating that GPR143 expressed in vascular smooth muscle cells could regulate α_1_ARs -mediated arterial contraction. In GPR143-KO smooth muscle cells, phenylephrine-induced phosphorylation of myosin light chain 2 (MLC2) and ERK phosphorylation were attenuated compared to *Wt* smooth muscle cells. MLC2 and ERK phosphorylation are causally related to phenylephrine-induced contraction, an effect involving GPR143. Pretreatment with l-DOPA at low nanomolar concentrations augmented the vasoconstricting effect of phenylephrine in isolated arteries from *Wt* but not GPR143-KO mice. Consistently, exogenously applied l-DOPA at 1 to 10 nM augmented the contractile and intracellular Ca^2+^ responses to phenylephrine in smooth muscle cells. The binding affinity of phenylephrine against the specific binding of [^3^H]-prazosin, an α_1_ARs antagonist, was higher in HEK293 cells coexpressing both α1_B_AR-Myc and GPR143-EGFP than in cells coexpressing α_1B_AR-Myc and free-EGFP. Immunoprecipitation assay also revealed that the interaction between GPR143 and α_1B_AR was enhanced by l-DOPA. Importantly, the enhancement was observed at a concentration comparable to those of plasma l-DOPA (10–20 nM) (Chritton et al., 1997; Goldstein et al., 2003; Masukawa et al., 2017). GPR143, when functionally coupled with α1_B_AR, may have significantly more affinity for l-DOPA than when not forming heteromers with α1_B_AR (Masukawa et al., 2017). Förster resonance energy transfer signal was detected in live HEK293 cells expressing α1_B_AR-Venus and GPR143-CFP, but not in those expressing vasopressin receptor AVPR1_A_ -CFP and GPR143-Venus (Masukawa et al., 2017). The direct interaction between GPR143 and α_1B_AR was demonstrated in *Wt* tissue by *in situ* proximity ligation assay. Furthermore, the stress-induced pressor response and the rise in blood pressure in active phase in GPR143-KO were blunted when compared to *Wt* mice. Together, these findings indicate that l-DOPA sensitizes contractile response to sympathetic outflow through coupling of GPR143 with αARs in vascular smooth muscle cells (Masukawa et al., 2017; **Figure 1**) and further suggest the physiological relevance of GPR143 as an l-DOPA receptor.

Recent findings indicate that the interaction between distinct GPCRs, forming hetero-oligomers, can affect affinity for agonists, the performance, and range through which extracellular signals are transmitted to G protein molecules (Ferre et al., 2014; Ferre, 2015; Farran, 2017). The functional interaction of hetero-oligomer GPCRs was observed for DA D_1_–D_3_ heterodimer receptors. D_1_–D_3_ receptor heterodimer had greater affinity for DA than did DA_1_ monomeric receptors without DA_3_ receptors (Fiorentini et al., 2008). Coupling between GPR143 and α1_B_AR could also occur in the central nervous system, because α1_B_AR was present in the nucleus accumbens and colocalized with dopamine D_1_ receptor (Mitrano et al., 2018). These finding suggest that some of the l-DOPA actions may be mediated through possible functional coupling between these GPCRs: D1, α1_B_AR and GPR143. l-DOPA itself did not interact with adrenergic α-, β-, D_1_, and D_2_ receptors (Goshima et al., 1991b). However, l-DOPA actions that were suppressed by antagonists for these receptors have been reported. For example, prazosin attenuated l-DOPA–induced hyperactivity without affecting its anti-PD actions or dyskinesia in 1-methyl-4-phenyl-1,2,3,6- tetrahydropyridine (MPTP)–lesioned macaques (Visanji et al., 2009). l-DOPA produced propranolol (nonselective β-adrenoceptor antagonist)–facilitatory action on the NA release from brain slices (Goshima et al., 1986). Propranolol attenuated l-DOPA–induced efflux of DA from the 6-OHDA–lesioned striatum and reduced l-DOPA–induced dyskinesia (Bhide et al., 2015).

Although GPR143 is the most plausible l-DOPA receptor candidate, we found that l-DOPA induced ptosis in GPR143-KO as well as *Wt* mice pretreated with a central AADC inhibitor (Ueda et al., 2016). Ptosis sometimes associates with short- or long-term l-DOPA treatment (Giladi et al., 1992). These findings raise the possibility that l-DOPA induces actions through both GPR143-dependent and GPR143-independent mechanisms as well as its conversion to DA.

## Pathophysiological Significance of l-DOPA/GPR143 Interaction in PD

Parkinson’s disease is characterized by neuronal loss of dopaminergic neurons, and enzymatic activities of TH and AADC decreased during the progression of the pathology (Lloyd et al., 1975; Nagatsu and Nagatsu, 2016). As mentioned, l-DOPA can induce orthostatic hypotension in the treatment of PD (Connolly and Lang, 2014; De Pablo-Fernandez et al., 2017). Thus, the reduction of TH levels and its subsequent decrease in the synthesis and release of endogenous l-DOPA may contribute to autonomic dysfunctions observed in PD (De Pablo-Fernandez et al., 2017). The possible reduction in endogenous levels of l-DOPA might be related to supersensitization of responses to l-DOPA or DA agonists in PD or experimental PD models (Ueda et al., 1995a; Shimamura et al., 2006; Bhide et al., 2015). On the other hand, long-term l-DOPA treatment could induce down-regulation or desensitization of the receptor(s) for l-DOPA. Thus, changes in GPR143 properties (expression, sensitization) may also arise in PD patients together with the progression of the pathology, which may account for the development of the side effects or loss of efficacy of l-DOPA.

The decrease in the AADC activity may also affect the pharmacological actions of l-DOPA. For example, in intact rat brain slices, l-DOPA increased the release of DA at 0.1 µM but produced no effect at 1 µM. In the presence of an AADC inhibitor, l-DOPA (1 µM) inhibited the release of DA (Goshima et al., 1986). Likewise, l-DOPA at 30 nM increased the DA release from intact slices, but decreased the release from the slices of MPTP-treated mice (Goshima et al., 1991a). These results imply that the action of l-DOPA can become predominant over the action of DA during the progression of PD. Another example is the ability of l-DOPA but not DA to induce the release of glutamate from the striatum (Goshima et al., 1993). The respective inhibitory and facilitatory effects of l-DOPA on the release of DA and glutamate seen under the decreased AADC activities (Goshima et al., 1986; Goshima et al., 1993) may be relevant to some adverse effects such as the decreased therapeutic efficacy, dyskinesia, or “on-off” encountered during long-term therapy (Marsden, 1994). The decreased AADC activities may further tip the balance in favor of the l-DOPA actions, because DA may act as an agonist or inverse agonist against GPR143 (Lopez et al., 2008).

Previous clinical data have suggested that l-DOPA either slows the progression of PD or has a prolonged effect on PD symptoms (Fahn et al., 2004). In contrast, a recent trial concluded that treatment with l-DOPA had no disease-modifying effect, either beneficial or detrimental, on early PD among patients who were evaluated over the course of 80 weeks (Verschuur et al., 2019). l-DOPA, DA, and related compounds have the potential to be cytotoxic, since free radical and quinone metabolites of l-DOPA were shown to be toxic *in vitro* and *in vivo* (Graham et al., 1978; Cohen, 1987; Smith et al., 1994). Our demonstration of increased glutamate release by l-DOPA (Goshima et al., 1993) provides *in vivo* evidence for a mechanism that could accelerate the degeneration process. On the other hand, it was shown that chronic l-DOPA promoted recovery in remaining DA neurons, increased mRNA, and induced release of BDNF in the brain of PD models (Murer et al., 1998; Zhang et al., 2006). Thus, specific assays suggest the possibility that l-DOPA might have both beneficial and detrimental effects depending on various conditions such as therapeutic doses of l-DOPA, its metabolism, and the degree of degeneration of DA neurons. Whether GPR143 is required for any long-term effects of l-DOPA is an important issue to be answered in future studies.

**Figure 2:**
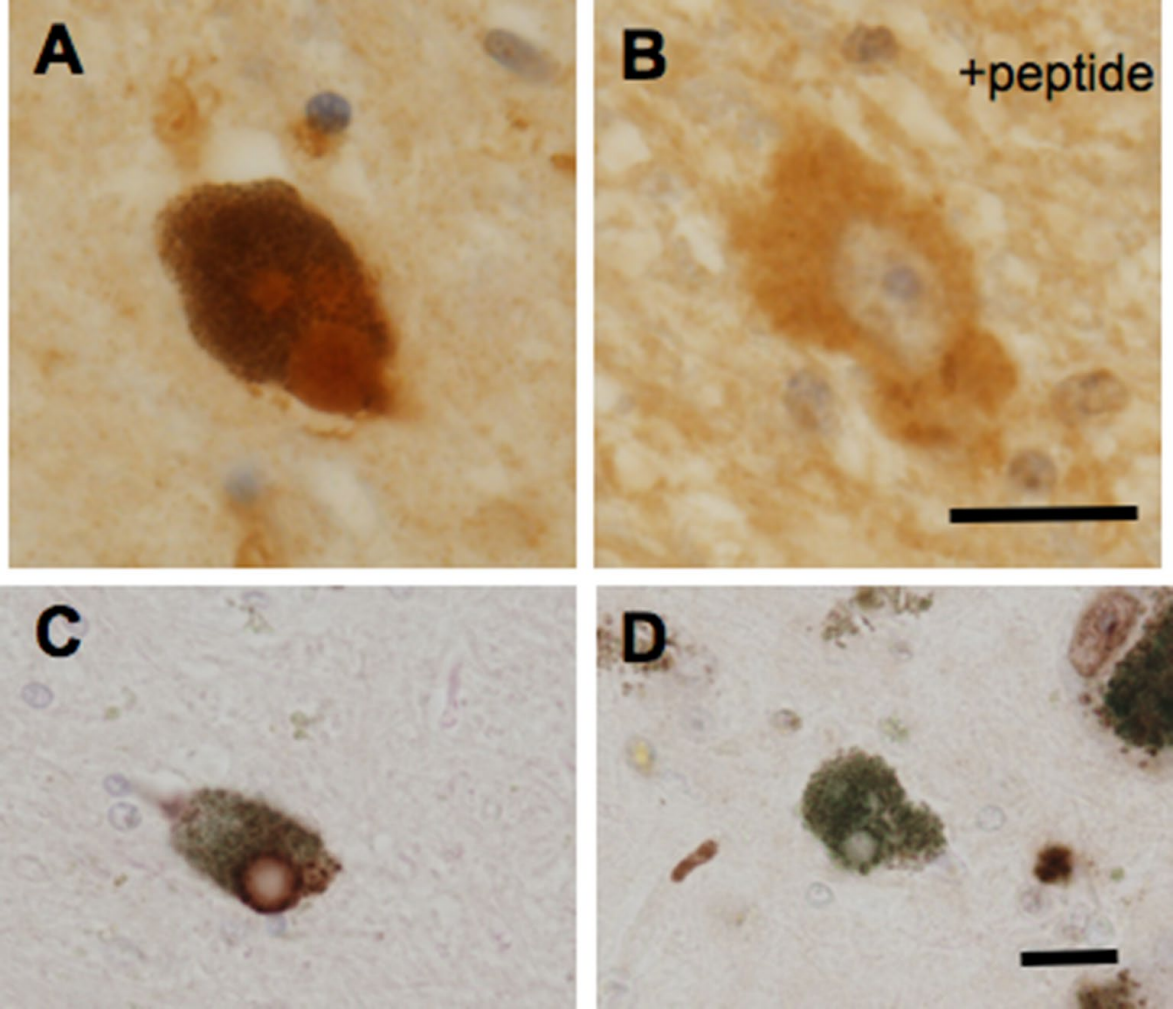
GPR143-positive **(A**, **C)** and GPR143–negative **(D)** Lewy bodies in neurons in the substantia nigra compacta of PD brain tissue. GPR143-positive signals were blocked by the synthetic peptide **(B)**. Scale bar, 20 μm. (Goshima et al., 2018).

**Figure 3:**
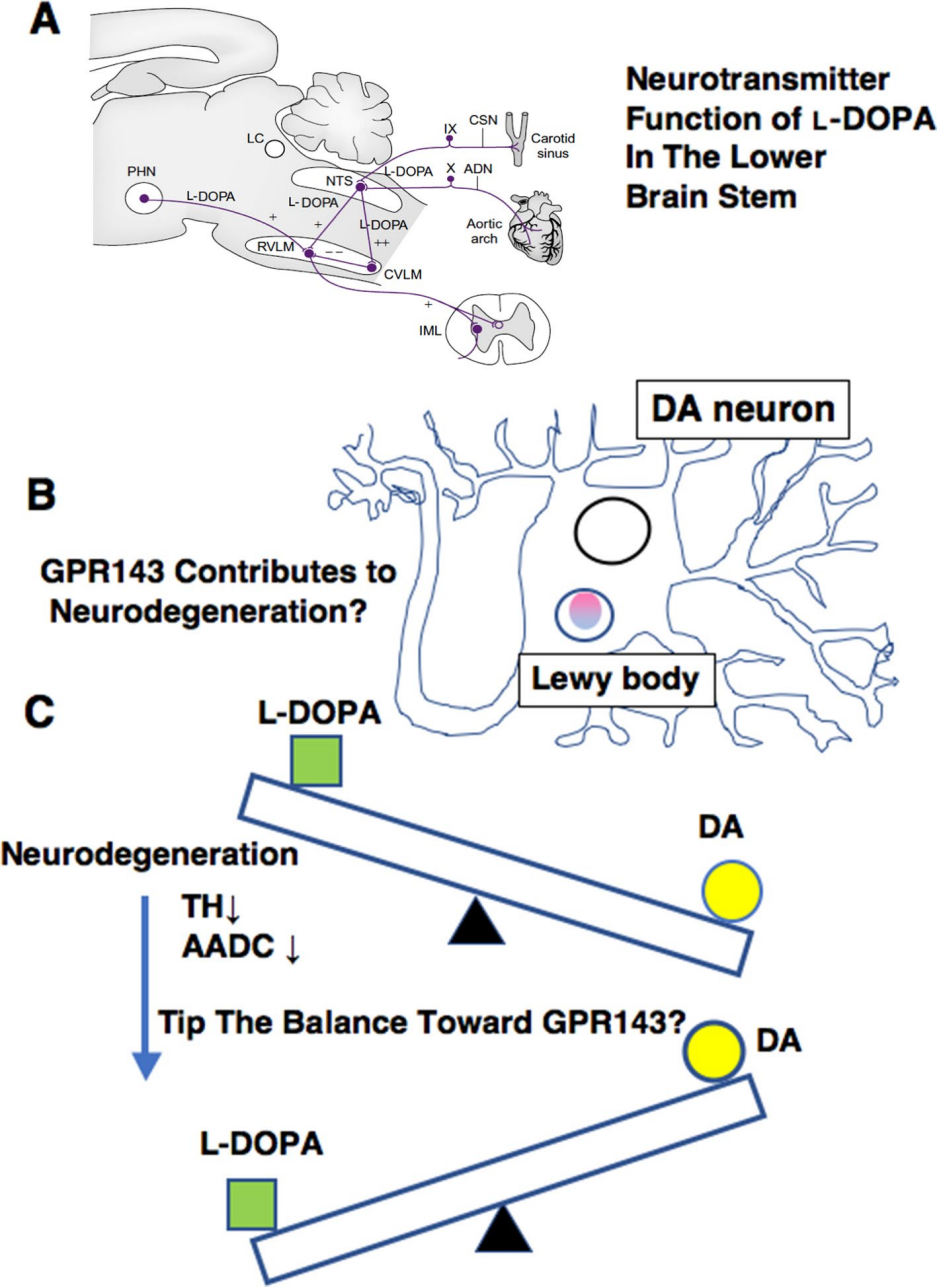
Summary of various ways in which l-DOPA may act for normal physiology **(A)**, for PD pathology **(B)**, and for PD treatment **(C)**. **(A)** l-DOPA may play a role in carrying baroreceptor information as a neurotransmitter of the primary baroreceptor afferents terminating in the NTS. ADN, aortic depressor nerve; CSN, carotid sinus nerve; CVLM, caudal ventrolateral medulla; IML, intermediolateral cell column; LC, locus caeruleus; NTS, nucleus tractus solitarii; PHN, posterior hypothalamic nucleus; RVLM, rostral ventrolateral medulla. (See details in Misu et al., 2002) **(B)** GPR143 might be related to PD pathogenesis. **(C)** A possible impact on l-DOPA therapy. The decrease in the AADC activity may affect l-DOPA/DA receptor signaling balance in PD brains.

In addition to a possible role of GPR143 in symptomatic-modifying effect of l-DOPA, GPR143 might also be involved in the pathogenesis of PD. To gain an insight into this issue, immunohistochemical analysis was conducted of PD brain tissues by using human anti-GPR143 antibody (Goshima et al., 2018). GPR143-immunoreactive neurons with large perikarya and neurites were observed in the midbrain of control and PD brains. The GPR143-immunoreactive signals showed a dot-like pattern in the perikarya, reflecting its localizations to melanosomes or late endosomes/lysosomes (Schiaffino, 2010). Consistently, unlike other GPCRs, an unconventional dileucine motif and a tryptophan-glutamic acid doublet motif were identified in GPR143 within its third cytosolic loop and the C-terminal tail, respectively, both of which may be responsible for driving its intracellular targeting (Piccirillo et al., 2006). In the PD brain tissue, GPR143 was colocalized with phosphorylated α-synuclein in Lewy bodies (Goshima et al., 2018) (**Figure 2**). It might be possible that accumulation and localization of GPCRs such as GPR143 are related to the pathogenesis of PD or to efficacy and/or untoward effects of l-DOPA. Interestingly, Parkin-associated endothelin receptor-like receptor (Pael-R) (GPR37) shared some properties common with those of GPR143. GPR37 showed overlapping expression pattern with GPR143 and poor trafficking to the plasma membrane. GPR37 was localized in Lewy bodies (Yang et al., 2003; Murakami et al., 2004) and, like GPR143, possessed high basal activities. Interestingly, overexpression of GPR37 resulted in death of dopaminergic neurons (Leinartaite and Svenningsson, 2017), while GPR37 was contributed to the signaling of certain neuroprotective factors (Meyer et al., 2013). Future studies should address whether GPR143 is also involved in the pathogenesis and the toward and/or untoward actions of l-DOPA in PD (**Figure 3**).

## Conclusion

l-DOPA is likely to play a role as a neurotransmitter as well as a precursor of DA. Some critical questions, however, remain unanswered. Does l-DOPA release occur by a vesicular or nonvesicular mechanism? How does GPR143 mediate l-DOPA actions at a single-cell level? Is GPR143 involved in the pathogenesis of PD? Is GPR143 the only functional receptor for l-DOPA? This is the beginning of a new era for the mechanistic study of l-DOPA as a neurotransmitter, its neuronal network specificity, and its pathogenic role, all of which surely have a major impact on l-DOPA therapy in PD.

## Ethics Statement

All the experiments were performed in accordance with the guidelines of ethical committees of Yokohama City University, Tokyo Metropolitan Neurological Hospital and Tokyo Metropolitan Institute of Medical Sciences. Informed consent was obtained from all subjects.

## Author Contributions

YG, DM, YK, TH and AC all contributed to the writing and editing of this review.

## Funding

YG is funded by a Grant-in-Aid for Scientific Research (B) (General) (no. 15H04687), Scientific Research (B) (General) (no. 18H02580), the Japanese SRF Grand for Biomedical Research (no. 1565), Uehara Memorial Foundation (no. 201320161 and 201720339), and the fund for Creation of Innovation Centers for Advanced Interdisciplinary Research Areas Program in the Project for Developing Innovation Systems from MEXT (no. 42890001). DM is funded by a Grant-in-Aid for Young Scientists (B) (no. 18K14923) and by the Uehara Memorial Foundation (201810115). TH is funded by a Grant-in-Aid for Scientific Research (C) (General) (no. 18K06896). YK is funded by a Grant-in-Aid for Young Scientists (no. 19K16375). AC is supported by Japanese government (MEXT) scholarship for PhD (no. 8513).

## Conflict of Interest

The authors declare that the research was conducted in the absence of any commercial or financial relationships that could be construed as a potential conflict of interest.
